# Productive Aging Across Nations and Cultures

**DOI:** 10.1093/geroni/igaf122.441

**Published:** 2025-12-31

**Authors:** Patrick Ho Lam Lai, Nancy Morrow-Howell

## Abstract

Engagement in productive activities such as employment and volunteerism is essential for well-being and societal development in later life, particularly while the aging population continues to grow across different cultures and countries. However, participation in these activities varies due to cultural, economic, social, and policy factors. This symposium examines productive aging from an international and cross-cultural perspective, highlighting disparities in engagement and well-being among older adults across various cultural and national contexts. The first presentation explores how culture-related factors such as family expectations and religion shape volunteerism among older Asian adults in the United States compared to other ethnoracial groups. The second presentation examines financial disparities among older self-employed workers in Sweden, comparing limited liability company owners, sole proprietors, and employees in terms of household income and social benefits. The third presentation analyzes gender disparities in depression among older adults from a cross-national and longitudinal perspective, considering the role of work and volunteerism while accounting for cohort and age effects. The final presentation analyzes disparities in U.S. older workers’ access to employer-provided health insurance, retirement savings, and flexible work arrangements by gender, race, and ethnicity, with a particular focus on Hispanic and Black workers. By exploring these diverse perspectives, this symposium advances understanding of the contextual influences on later-life engagement. The discussant will synthesize key insights and discuss implications for policies and programs that promote equitable participation in productive aging across cultures and countries.

